# The Positive Choices trial: study protocol for a Phase-III RCT trial of a whole-school social marketing intervention to promote sexual health and reduce health inequalities

**DOI:** 10.1186/s13063-021-05793-6

**Published:** 2021-11-17

**Authors:** Ruth Ponsford, Rebecca Meiksin, Elizabeth Allen, G. J. Melendez-Torres, Steve Morris, Catherine Mercer, Rona Campbell, Honor Young, Maria Lohan, Karin Coyle, Chris Bonell

**Affiliations:** 1 London School of Hygiene and Tropical Medicine, 15-17 Tavistock Place, WC1H 9SH London, UK; 2 London School of Hygiene and Tropical Medicine, Keppel Street, WC1E 7HT London, UK; 3 University of Exeter College of Medicine and Health, South Cloisters, St Luke’s Campus, Heavitree Road, EX1 2 Exeter, LU UK; 4 Department of Public Health & Primary Care, Cambridge University, Strangeways Research Laboratory, Worts Causeway, Cambridge, CB1 8RN UK; 5 UCL Institute for Global Health, 3rd Floor Mortimer Market Centre, off Capper Street, WC1E 6JB London, UK; 6 University of Bristol, 1-5 Whiteladies Road, Clifton, Bristol, BS8 1NU UK; 7 DECIPHer, Cardiff School of Social Sciences, Cardiff University, 1-3 Museum Place, Cardiff, CF10 3BD UK; 8 School of Nursing and Midwifery, Medical Biology Centre, 97 Lisburn Road, Belfast, BT9 7BL UK; 9 5619 Scotts Valley Drive, Suite 140, Scotts Valley, CA 95066 Scotts Valley, USA

**Keywords:** Schools, Adolescents, Sexual health, Sexual competence, Pregnancy, Sexually transmitted infections, Whole-school, Cluster trials, Public health

## Abstract

**Background:**

Positive Choices is a whole-school social marketing intervention to promote sexual health among secondary school students. Intervention comprises school health promotion council involving staff and students coordinating delivery; student survey to inform local tailoring; teacher-delivered classroom curriculum; student-run campaigns; parent information; and review of sexual/reproductive health services to inform improvements. This trial builds on an optimisation/pilot RCT study which met progression criteria, plus findings from another pilot RCT of the Project Respect school-based intervention to prevent dating and relationship violence which concluded such work should be integrated within Positive Choices. Young people carry a disproportionate burden of adverse sexual health; most do not report competence at first sex. Relationships and sex education in schools can contribute to promoting sexual health but effects are small, inconsistent and not sustained. Such work needs to be supplemented by ‘whole-school’ (e.g. student campaigns, sexual health services) and ‘social marketing’ (harnessing commercial marketing to social ends) approaches for which there is good review-level evidence but not from the UK.

**Methods:**

We will conduct a cluster RCT across 50 schools (minimum 6440, maximum 8500 students) allocated 1:1 to intervention/control assessing outcomes at 33 months. Our primary outcome is non-competent first sex. Secondary outcomes are non-competent last sex, age at sexual debut, non-use of contraception at first and last sex among those reporting heterosexual intercourse, number of sexual partners, dating and relationship violence, sexually transmitted infections, and pregnancy and unintended pregnancy for girls and initiation of pregnancy for boys. We will recruit 50 school and undertake baseline surveys by March 2022; implement the intervention over the 2022–2024 school years and conduct the economic and process evaluations by July 2024; undertake follow-up surveys by December 2024; complete analyses, all patient and policy involvement and draft the study report by March 2025; and engage in knowledge exchange from December 2024.

**Discussion:**

This trial is one of a growing number focused on whole-school approaches to public health in schools. The key scientific output will be evidence about the effectiveness, costs and potential scalability and transferability of Positive Choices.

**Trial registration:**

ISRCTN No: ISRCTN16723909. Trial registration summary: Date:. Funded by: National Institute for Health Research Public Health Research Programme (NIHR131487). Sponsor: LSHTM. Public/scientific contact: Chris Bonell. Public title: Positive Choices trial. Scientific title: Phase-III RCT of Positive Choices: a whole-school social marketing intervention to promote sexual health and reduce health inequalities. Countries of recruitment: UK. Intervention: Positive Choices. Inclusion criteria: Students in year 8 (age 12–13 years) at baseline deemed competent by schools to participate in secondary schools excluding pupil referral units, schools for those with special educational needs and disabilities, and schools with ‘inadequate’ Ofsted inspections. Study type: interventional study with superiority phase III cluster RCT design. Enrollment: 1/9/21-31/3/22. Sample size: 50 schools and 6440–8500 students. Recruitment status: pending. Primary outcome: binary measure of non-competent first sex. Secondary outcomes: non-competent last sex; age at sexual debut; non-use of contraception at first and last sex; number of sexual partners; dating and relationship violence (DRV) victimisation; sexually transmitted infections; pregnancy and unintended pregnancy for girls and initiation of pregnancy for boys using adapted versions of the RIPPLE measures. Ethics review: LSHTM research ethics committee (reference 26411). Completion data: 1/3/25. Sharing statement: Data will be made available after the main trial analyses have been completed on reasonable request from researchers with ethics approval and a clear protocol. Amendments to the protocol will be communicated to the investigators, sponsor, funder, research ethics committee, trial registration and the journal publishing the protocol. Amendments affecting participants’ experience of the intervention or important amendments affecting the overall design and conduct of the trial will be communicated to participants.

## Background

### Overview

This protocol is for a phase-III cluster randomised controlled trial (RCT) of Positive Choices, a whole-school social marketing intervention to promote sexual health and reduce health inequalities in English secondary schools. It builds on an earlier optimisation, feasibility testing and pilot RCT study of Positive Choices, as well as another pilot RCT of the Project Respect intervention to prevent dating and relationship violence (DRV). Within the Positive Choices pilot study, we first optimised and tested the feasibility of the intervention in collaboration with the Sex Education Forum (SEF) and one secondary school, with additional public and policy involvement (PPI). We then conducted a pilot RCT across six schools, which met all the pre-specified criteria for progression to a phase-III RCT. The Project Respect pilot study concluded that prevention of DRV should be integrated within broader relationships and sex education (RSE) and so this phase III protocol incorporates such provision within the Positive Choices curriculum. The study will be the first UK RCT of an intervention to promote adolescent sexual health using whole-school and social marketing approaches, for both of which there is evidence of effectiveness from international studies.

### Adolescent sexual health

The Positive Choices intervention aims as its primary outcome to improve sexual competence at first sex. This has been defined through the National Survey of Sexual Attitudes and Lifestyles (Natsal) in terms of the following at first sex: use of contraception; autonomy of decision to have sex (not due to drunkenness, external pressure, etc.); partners being equally willing to have sex; and individuals judging it to have been the ‘right time (not reporting they should have waited longer, etc.)’; see uploaded set of items. Most young people in Britain do not report competence at first sex [1]. Lack of competence at first sex is strongly associated with increased risk across adolescence and adulthood of unplanned pregnancy, sexually transmitted infection (STI) diagnosis (ever) among young women, experiencing non-volitional sex (ever) and sexual function problems (in the last year) [2]. Lack of competence at first sex is a stronger predictor of adverse sexual health outcomes than age of sexual debut alone, particularly among young women and particularly for a broader range of outcomes including non-volitional sex [2].

In England and Wales, young people carry a disproportionate burden of adverse sexual health with some risks increasing by year. Age of sexual debut is becoming earlier [3, 4]. Rates of STIs among the population overall and among young people aged 15–24 years are also increasing [5]. There were 447,694 diagnoses of STIs in England in 2018, a 5% rise since 2017. There were 56,259 diagnoses of gonorrhoea, a 26% rise since 2017. The burden of STIs is greatest among young people aged 15–24 years. Young people in the UK also experience high rates of non-volitional sex, sexual violence and dating and relationships violence (DRV) compared to adults in the UK and youth in other countries [6–9]. Recent surveys of young people in England suggest that, among those aged 14–17 in England who have been in a relationship, 22% of girls report physical DRV, 48% report emotional DRV (offline), 48% report emotional DRV (online) and 41% report sexual DRV. Among boys, these figures were 12%, 27% (online), 25% (offline) and 14% [10]. Early experience of DRV is associated with subsequent adverse outcomes such as STIs and mental health problems [11, 12]. The UK still has the highest rate of teenage births in western Europe and teenagers remain the age group at highest risk of unplanned pregnancy. Despite a decline of 64% from 1998, in 2017, the under-18 conception rate in England and Wales was 16.8 conceptions per thousand women aged 15–17 [13]. Even after controlling for prior disadvantage, teenage pregnancy is associated with adverse medical, social, educational and economic outcomes for both mothers [14–16] and children [17, 18]. Teenage pregnancy is subject to and contributes to maintaining health inequalities [19]. The cost for 2013–2020 of teenage pregnancy and STIs in the UK is estimated as £84.4 to 127 billion, aggregating NHS and broader public-sector costs [20]. There are considerable returns on investment for prevention interventions particularly among adolescents [21].

### Interventions to promote adolescent sexual health

There is good evidence that RSE delivered in school classrooms can contribute to promoting sexual health, and preventing unintended pregnancies and STIs [22–27]. Features associated with effective RSE interventions include the following: addressing individual knowledge, attitudes, self-efficacy and skills; addressing gender and other social norms; and use of interactive, culturally appropriate methods and materials [22–29]. Informed by this evidence, Positive Choices is informed by an explicit theory of change to ensure it systematically addresses knowledge, attitudes, self-efficacy, skills and social norms; and uses interactive, culturally relevant methods.

However, reviews also suggest that the effects of classroom-based RSE are often small, inconsistent between studies and generally not sustained over time. This suggests the need for classroom RSE to be supplemented by other approaches, such as ‘whole-school’ approaches that aim to build student engagement with school, supportive social norms and better access to sexual and reproductive health services in or near schools. There is good evidence from existing reviews that whole-school interventions are effective in delaying sexual debut, increasing contraception use and preventing STIs and teenage pregnancy [28, 30–32]. Informed by this evidence, Positive Choices comprises multiple components addressing classroom and whole-school environments and increases student access to local sexual and reproductive health services.

Furthermore, there is increased policy and scientific interest in social marketing interventions to promote adolescent sexual health [33]. Social marketing aims to achieve social benefits using methods adapted from commercial marketing. A recent systematic review of social marketing interventions to reduce teenage pregnancy suggested that these are a particularly promising strategy. This review examined studies of interventions embracing social marketing elements [34] regardless of whether these were explicitly termed ‘social marketing’ [35]. Heterogeneity precluded meta-analysis but narrative synthesis found consistent evidence of effectiveness across outcomes and studies [35]. Positive Choices is informed by the three social marketing interventions with the strongest international evidence of effectiveness: Safer Choices, the Children’s Aid Society Carrera programme and the Gatehouse Project.

Safer Choices is a US school-based social marketing intervention involving the following: a school health promotion council coordinating intervention activities; a classroom-based sexual health curriculum; social-marketing campaigns formulated and implemented by students; and information for parents. An RCT of this intervention reported multiple sexual health benefits [36–38]. Sexually experienced students in intervention schools reported less frequent intercourse without a condom than controls (*P* = 0.05) by a ratio of adjusted means of 0.63. Safer Choices students also reported fewer partners with whom they had unprotected sex than controls (*P* = 0.02) by ratio of means of 0.73. Safer Choices students were more likely to use condoms (OR = 1.68; *P* = 0.04), and more likely to use effective contraception (OR = 1.76; *P* = 0.05) than controls. Informed by this evidence, Positive Choices includes a school health promotion council to coordinate delivery, a classroom curriculum for years 9 and 10 (age 13–15), social marketing campaigns run by students to promote sexual health and parent information addressing parent-child communication as a determinant of sexual health.

The Children’s Aid Society Carrera programme is a US after-school intervention providing education, life-skills training and links to sexual and reproductive health services. An RCT of this intervention in New York City reported fewer pregnancies (OR = 0.31, *P* < 0.01), delayed sexual debut (OR = 0.52, *P* < 0.05) and increased use of effective contraception at last sex (OR = 2.37, *P* < 0.05) among girls [39]. Informed by this evidence, Positive Choice’s classroom curriculum addresses social and emotional life-skills alongside sexual health content and aims to increase young people’s access to school-based and/or local sexual and reproductive health services.

The Gatehouse project is an Australian whole-school intervention which includes a student needs survey, a student/staff decision-making group coordinating whole-school actions and classroom-based curriculum addressing social and emotional learning. An RCT in high schools in Victoria reported participants’ increased age at sexual debut (OR = 0.55, 95% confidence interval 0.37–0.83) [40]. Informed by this evidence, Positive Choices uses a student needs survey to inform decisions by the school health promotion council (which includes student and staff members) and teaches social and emotional skills as described above. No studies in the review of social marketing interventions were conducted in the UK, and until Positive Choices, there have been no UK trials of school-based social marketing interventions to promote sexual health. This is an important gap because it cannot be assumed that sexual health interventions found to be positive elsewhere are automatically transferable to the UK [41] particularly given international differences in school systems and capacities, as well as young people’s social norms, hence the need for this trial.

### Results of the optimisation and pilot RCT study

Intervention optimisation involved collaboration with one secondary school, and PPI with the Advice Leading to Public Health Action (ALPHA) group of young people based at the DECIPHer Centre in Cardiff, trained to provide advice on public health research, as well as a group of policy/practice stakeholders. This elaborated Positive Choices from a basic description to a fully specified intervention with materials. This was successful and all components were successfully feasibility-tested in the collaborating school. The curriculum was refined and broadened from that originally planned to ensure that it provided comprehensive RSE while still addressing all topics in the protocol. Social marketing campaigns segmented student audiences only on age, informed by PPI with teachers and students suggesting that other targeting would stigmatise those students. Review of sexual and reproductive health services was reoriented away from what was originally planned as SEF consultancy for schools, towards a guided school self-audit to increase cost-efficiency and school capacity for sustainability.

In the subsequent pilot RCT across 6 schools, all criteria for progression to phase III were met. All schools were randomised, 4 to intervention and 2 to control, and remained in the study. Student response rates in intervention and control groups were 89% and 84% at baseline, and 89% and 82% at follow-up. The intervention was implemented with fidelity in 3 intervention schools with limited data from 1 school preventing assessment of fidelity. Around two-thirds of students reported awareness of the intervention, and around 80% of these students and all staff interviewed indicated acceptability. Positive Choices students reported significantly more comprehensive coverage of RSE topics than controls. Linkage of self-report and registered births and terminations was feasible but, unsurprisingly, given students’ ages and national trends, there were no registered births or terminations among trial participants.

Measures were assessed as reliable and economic evaluation methods were feasible. Staff interviews suggested that senior leadership team commitment was critical to successful delivery. Schools struggled with the short time between being allocated to the intervention group (July in the pilot) and then being required to start delivering the intervention (September in the pilot). To increase efficiency and school capacity to sustain the intervention, a train-the-trainer model (with SEF training selected school staff to train other staff) was recommended. Students reported Positive Choices enabled more open conversations about sexual health and raised awareness of sexual rights and responsibilities. Staff reported that the intervention enhanced student engagement. Parents found the intervention acceptable. No student harms were apparent.

PPI with youth and policy/practitioner stakeholders, and consultation with the study steering committee (SSC) supported the view that Positive Choices should now be subject to a phase III RCT. Stakeholder workshops suggested considerable interest in whole-school and social marketing interventions as a means to implement new statutory requirements for schools to implement relationships, sex and health education. PPI with policy/practice stakeholders recommended a longer lead-in from allocation to intervention and delivery, intervention costs being met by schools or local authorities and including a focus on DRV as part of the intervention. PPI with the policy/practice stakeholders and the ALPHA youth group also recommended a change in the primary outcome from pregnancy to lack of sexual competence at first sex. The rationale was that while teenage pregnancy rates are declining, other risks such as STIs and DRV are increasing; lack of sexual competence at first sex is an excellent indicator of overall sexual risk that is strongly associated with subsequent risk in adolescence or adulthood of STI among young women, unplanned pregnancy, ever having experienced non-volitional sex and sexual dysfunction [2]; and lack of sexual competence at first sex is relevant to all young people regardless of gender and sexual orientation whereas pregnancy is applicable only to heterosexual intercourse among girls.

### Changes to intervention and research methods for phase III protocol

The original pilot RCT protocol made clear that in the pilot the curriculum would only target students in year 9, whereas in phase III the curriculum will target students as they moved from year 9 into year 10 (and this remains our plan). In the phase III RCT, the intervention will also address DRV, informed by our consultation with policy/practice stakeholders in the pilot RCT, and by our finding from the pilot RCT of Project Respect to prevent DRV that this outcome is best addressed as part of a broader RSE intervention rather than through a stand-alone intervention [42]. The incorporation of DRV content into Positive Choices is supported by evidence that sexual health education can effectively reduce DRV and sexual assault [43]. To address DRV, existing additional content on DRV will be incorporated into the Positive Choices curriculum. This element from Project Respect was informed by evidence of effective DRV prevention, notably the Safe Dates and Shifting Boundaries trials [44, 45]. As recommended in the Positive Choices pilot RCT, staff training will use a train-the-trainer model whereby school lead staff will be trained and cascade this to colleagues. Training on running school health promotion councils will be reoriented to consist of an extended workshop with senior leadership teams in each school to build commitment and ensure good planning in the start-up phase. We will also review methods and materials to assess need for refinements in the context of COVID-19. The production of the curriculum materials for year 10 and the integration of existing material on DRV into Positive Choices materials will be led by SEF and the research team in the start-up phase in collaboration with the ALPHA youth group and a policy/practice stakeholder group, with sign-off by the SSC.

Regarding research methods, the pilot found that schools would be more ready to deliver the intervention if there were a longer period from random allocation to delivery. We therefore plan a start-up phase between notifying schools whether they have been allocated to the intervention group (March 2022) and schools beginning to deliver the intervention to students (September 2022). During this phase, schools will be trained and prepare for delivery by planning timetabling and staffing of lessons and other activities.

The primary outcome piloted in the pilot RCT was pregnancy assessed via registered births and terminations. However, pregnancy does not reflect the areas of greatest sexual health need in terms of current trends, and powering a trial on this outcome would require an unfeasibly large school sample [46]. The most recently reported prevalence of conceptions among girls aged 15–17 is 1.8% [13]. Furthermore, as raised by PPI stakeholders, this outcome is too narrow, focusing on one risk and relevant only for girls and heterosexual sex. Instead, we will use a self-reported measure indicating lack of competence at first sex as our primary outcome [1, 2], which was the strong preference of the ALPHA young researchers’ group and the policy/practice PPI stakeholder group. This outcome can be reliably measured and is strongly associated with subsequent multiple sexual health outcomes as described above [1, 2]. The measure was piloted in the Positive Choices pilot RCT where it performed well in terms of completion (88%) and inter-item reliability (ordinal alpha = 0.74). All of the above refinements are informed by the pilot studies of Positive Choices and Project Respect, do not reflect fundamental changes to intervention theory or approaches and so require no additional piloting.

### Rationale and aims

The above evidence indicates that Positive Choices meets a clear, evidenced, long-term need in terms of high prevalence of non-competence at first sex, STIs, unintended pregnancies, non-volitional sex, sexual violence and DRV, all of which are associated with major social and economic costs. Positive Choices is informed by previous systematic reviews [22–27] and by whole-school and social marketing interventions with strong evidence of effectiveness [36–38, 40, 44, 45, 47] and has been successfully piloted. The phase III RCT will be informed by learning from the pilot RCTs of the Positive Choices and Project Respect interventions, and be the first UK RCT of a whole-school social marketing intervention. This will generate evidence of scientific importance and policy relevance, addressing a key evidence gap as to whether whole-school social marketing interventions are an effective means of promoting sexual health in the English context. Relationships, sex and health education becoming statutory in English schools from September 2020 will add to the feasibility of recruitment and to the policy importance of the trial.

There are major potential public health benefits and potential benefits for participating individuals arising from the prevention of the adverse sexual health outcomes listed above. The pilot and previous studies suggest that participants are unlikely to experience increased sexual or other risks from the intervention or research [26]. In the unlikely event of the intervention generating harms, these would be identified via our surveys, qualitative research and monitoring of safeguarding concerns and serious adverse events (SAE) described below. We will maximise retention, minimise disruption to schools and ensure data quality by employing strategies we have previously used, such as close liaison between a named researcher and school day-to-day lead to identify convenient times and places for research, and identify problems early, and compensating schools for the costs arising from their participation in research activities.

The research aims to conduct a phase III RCT (50 schools) to examine the implementation, effectiveness, cost-effectiveness and mechanisms of the Positive Choices intervention and answer the following research questions:

1. What is the effect of the Positive Choices intervention in intention-to-treat analyses on student-reported measures of non-competent first sex (primary outcome), and various pre-hypothesised secondary outcomes and intermediate outcomes?
2. Are these associations moderated by student gender, sexual orientation, ethnicity or socioeconomic status (SES), or by school-level GCSE attainment or local deprivation?
3. Are associations greater in on-treatment analyses accounting for intervention fidelity?
4. What does the intervention cost and is it cost-effective?
5. Is the intervention delivered with good fidelity, reach and acceptability and how does this vary between schools and students?
6. What is usual treatment in control schools?
7. What do qualitative data suggest about implementation processes/intervention mechanisms and how these might vary between schools or students?
8. What do the trial findings overall suggest about the intervention theory of change and the potential for the intervention to be delivered and be effective elsewhere?

### Objectives

- To recruit 50 schools for the RCT by December 2021.
- To undertake baseline surveys of students in year 8 (age 12/13 years) and randomise schools by March 2022.
- To refine the intervention so that it includes a year-10 curriculum and addresses other points identified in the pilot phase, informed by consultation with youth and policy/practice PPI stakeholders by July 2022.
- To prepare schools for implementation via start-up meetings and SEF and internal staff training by July 2022.
- To implement the intervention over the 2022/23 and 2023/24 school years by July 2024.
- To conduct the economic and process evaluations by July 2024.
- To undertake follow-up surveys of students at the start of year 11 (age 15/16) at 33 months post-baseline by December 2024.
- To analyse data to address the above research questions and draft the study report by 31 March 2025.
- Informed by consultation with youth and policy/practice PPI stakeholders, to engage in knowledge exchange and assess potential scale-up from December 2024.

## Methods

### Design

**Fig. 1:**
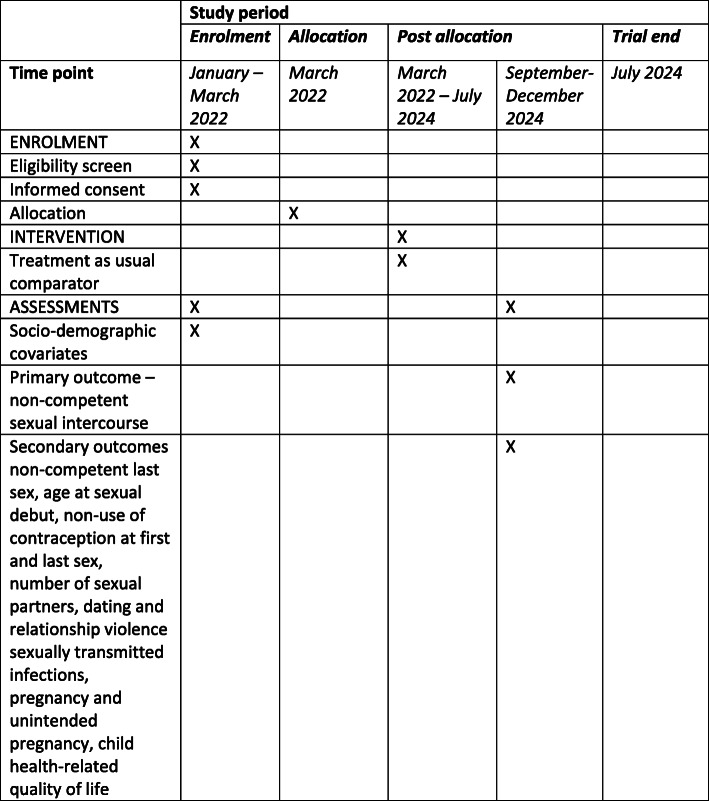
SPIRIT figure

**Fig. 2:**
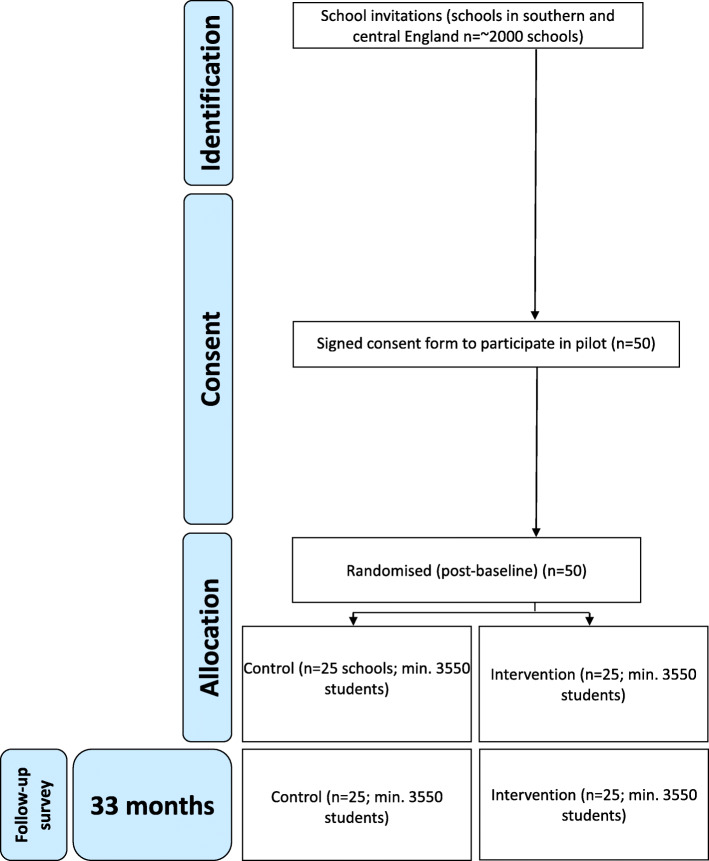
Positive choices RCT participant flow

We will conduct a superiority, phase III cluster parallel-group RCT to assess whether incorporating Positive Choices into school provision is more effective than usual provision (Fig. 1). The trial will comprise 50 schools (minimum 6440, maximum 8500 students) allocated 1:1 to intervention/control assessing primary and secondary outcomes at 33 months (Fig. 2) using primary intention-to-treat analyses, and using realist approaches to assessing contextual variations in implementation, mechanisms and outcomes.

### Settings

Positive Choices is intended to be deliverable in all English secondary schools (including faith schools, free schools, academies and private schools) excluding pupil referral units, schools for those with special educational needs and disabilities, and schools with ‘inadequate’ Ofsted inspections (which would lack the capacity to deliver until they gain an improved inspection report). This focus on all schools reflects the universal approach that the intervention takes, which recognises that population-level impacts on most sexual health outcomes cannot be achieved through targeted interventions because of the normal distribution of population risks [19, 48]. The trial will aim to recruit a representative (in terms of school type, GCSE attainment and Income Deprivation Affecting Children Index) sample of the above schools from across southern and central England.

### Study population

The study population is defined as students aged 12–16 years moving from year 8 into year 11 during the trial. The majority of these students will have received the intervention in years 9–10. This age group is targeted because of the following: proximal risk factors for adverse sexual health outcomes are manifesting [48], prevention is not too late; and RSE is acceptable [37, 49, 50]. PPI suggests provision to year 11 students is unfeasible because of GCSE preparation. Outcomes will be analysed among students reporting data at 33-month follow-up with any missing baseline data on socio-demographic characteristics or age of sexual debut being imputed based on responses at follow-up. No students deemed competent to consent by schools will be excluded from research recruitment and consent procedures unless parents withdraw them from the research. Those consenting to participate who have mild learning disabilities or limited English will be supported to complete the questionnaire by researchers.

### Analytic sample and sample size

We calculate that, for 80% power with 5% significance, conservatively assuming a sample of 140 students per school; drop out of 2 schools per arm; 80% student survey response rate at follow-up; an intra-cluster correlation coefficient (ICC) of 0.015 for our primary outcome; and prevalence of this outcome of 9% among those in the control arm, we will require 25 schools per arm to detect a 36% reduction in the primary outcome (giving a prevalence of 5.85% in the intervention arm). The minimum analytic sample for outcome assessment in the RCT will therefore be approximately 6440 students providing follow-up data at 33 months. Repeating this power calculation for the originally intended primary outcome of conceptions (where the prevalence among female controls would be around 2%) [13] would require > 100 schools per arm.

These estimates are informed by research and PPI. We assume 140 students per school informed by our recent INCLUSIVE trial [51]. This is a conservative assumption for the Positive Choices trial because the average year group has now risen to 190 and so assuming an 80% response rate (again informed by the Positive Choices pilot and previous studies [42, 51–53]) this would comfortably provide over 150 students [53]. Two schools dropping out is also a conservative assumption given our previous trials have experienced no drop outs [42, 51, 52]. Regarding ICCs, no data are available on school-level clustering of lack of sexual competence at first sex but we would expect this to be very similar to ICCs for non-use of contraception at first sex, which are reported as 0.01 [54, 55] so a conservative assumption of 0.015 for this study is reasonable.

Our assumption about prevalence of the primary outcome among controls is also informed by the literature. Around 25% of 15–16-year-olds are sexually active compared with 7% of 12–13-year-olds [56]. Therefore, between baseline and follow-up, we would expect 18% of students to become sexually active. Data from young people participating in the third Natsal sexual behaviour survey suggest that, among those aged 15–16 years, around half lack sexual competence at first sex [1, 2]. Therefore, we would expect around half of trial participants who become sexually active by follow-up (i.e. 9% of all participants) will experience non-competent sexual debut.

Finally, our assumed minimal effect on reducing non-competent first sex by 36% represents an effect of public health significance informed by consultation with policy stakeholders (personal communication Alison Hadley 2019). Furthermore, this magnitude of effect is in line with those reported for similar measures from trials of the interventions which have informed Positive Choices. The Safer Choices RCT reported less frequent intercourse without a condom among the intervention group than controls by a ratio of adjusted means of 0.63 (*P* = 0.05). As reported earlier, Safer Choices students were more likely to use condoms by an OR = 1.68 and more likely to use effective contraception by an OR = 1.76 than controls [36–38]. The Children’s Aid Society Carrera programme RCT reported increased use of effective contraception at last sex by an OR = 2.37 [39].

### Recruitment and randomisation

We will recruit 50 schools across central and southern England. As with our previous trials, schools will be recruited by a combination of emails and phone calls to schools, local authorities, school networks and academy chains, as well as a recruitment event and visits. Recruitment materials will indicate Positive Choice’s alignment with but greater coverage than statutory guidance plus its alignment with Ofsted criteria and safeguarding guidance. Response rates will be recorded, as will any stated reasons for non-participation. After baseline surveys with students in term 2 of year 8 (November 2021–March 2022), schools will be randomly allocated 1:1 to intervention/ control as a single batch remotely using a random number generator by LSHTM clinical trials unit (CTU), stratified by school-level GCSE attainment and local index of deprivation, which are key predictors of sexual health [48]. Schools will be given unique study numbers to preserve allocation concealment within the CTU. The fieldwork team will be informed of allocations and inform schools. Informed by the pilot and previous studies and in line with the ‘If I Were Jack’ RCT [57], we will incentivise recruitment and retention by offering a £500 payment to all schools with an additional £1000 for those allocated to the intervention group and a further £500 for case study schools, reflecting the greater research load on such schools. Our pilot and other studies suggest that payment is now absolutely essential for maintaining school participation in research [42, 52]. Each school will also be allocated a named research liaison contact to facilitate retention.

### Intervention and comparator

Positive Choices is a whole-school intervention with the following components informed by the Safer Choices, Children’s Aid Society Carrera and Gatehouse interventions:

1. School health promotion council comprising a diverse group of 6 staff (including RSE/Personal, Social, Health and Economic Education (PSHE) coordinator and senior leadership team members) and 6 students (diverse by school engagement and gender, inclusive by sexual orientation), which meets termly to plan, launch, coordinate and oversee delivery of components 3–6. Each school nominates an intervention champion (senior leadership team member) and a day-to-day intervention lead (RSE/PSHE coordinator) both of whom will sit on the school health promotion council, an approach successfully used in other secondary school interventions [58].
2. A student needs survey of year-8 students (age 12/13; drawing on the baseline RCT survey) which identifies areas of need overall (e.g. specific knowledge gaps, risk behaviours) and by student subgroups including disadvantaged and sexual/gender-minority students and provides evidence to help the school health promotion council build school commitment to the work and inform local tailoring of intervention components 3–6.
3. A classroom curriculum addressing social and emotional skills and relationships and sexual health knowledge and skills, delivered by school staff to increase scalability and sustainability. The curriculum is designed as a set of learning modules, some of which are delivered in all schools (8 lessons for year 9; 4 for year 10) and some of which are ‘add-on’ lessons, with schools choosing from a menu of lessons based on assessed student needs (2 for year 9; 1 for year 10) providing 10 h for year 9 and 5 h for year 10 in total. For year 9, essential lessons are (a) the female/male body and reproductive organs; (b) fertility and contraception; (c) STIs and safer sex; (d) building blocks to good relationships; (e) consent; (f) sustaining relationships; (g) sexual response and pleasure; and (h) pornography. ‘Add-on lessons’ are (i) pregnancy options; (ii) readiness for intimacy; (iii) body image and the digital world; (iv) female genital mutilation; and (v) human rights, stigma and discrimination. Overall, provision will align with statutory requirements.
4. Student-run social marketing campaigns facilitated by trained teachers and formulated and implemented by 12–18 students per school, diverse by gender and school engagement aiming to appeal to a diversity of students including disadvantaged and sexual/gender-minority students. Campaigns use social and other media, posters and events and focus for example on healthy relationships, sexual rights, DRV and/or access to local services, with at least one campaign being delivered per year.

(5) Parent information—three newsletters and two homework assignments per year addressing parent-child communication.

(6) Review of school and other local sexual and reproductive health services to inform improvements in provision and/or access.

Delivery of these components is supported by SEF providing schools with the following: a 2-h start-up meeting with each school’s senior leadership team and RSE coordinator to build commitment and enable timetabling and staffing; train-the-trainer sessions for selected school staff delivered via a blend of 7-h face-to-face (for curriculum) and 2-h online (for social marketing) training per year; a manual; materials for staff training, the curriculum and parent information; and guidance for the social marketing campaigns and sexual and reproductive health services review (all materials will be downloadable via a password-protected website). Curriculum training is then cascaded to classroom teachers in 2 × 3-h internal trainings per year. The intervention aims to modify some existing provision (RSE curriculum for years 9 and 10; parent information; access to local health services) as well as providing new activities (school health promotion council; needs survey; social marketing campaigns). Implementing the Positive Choices intervention will not require alteration to ‘treatment as usual’ teaching and these will continue for both trial arms.

**Fig. 3:**
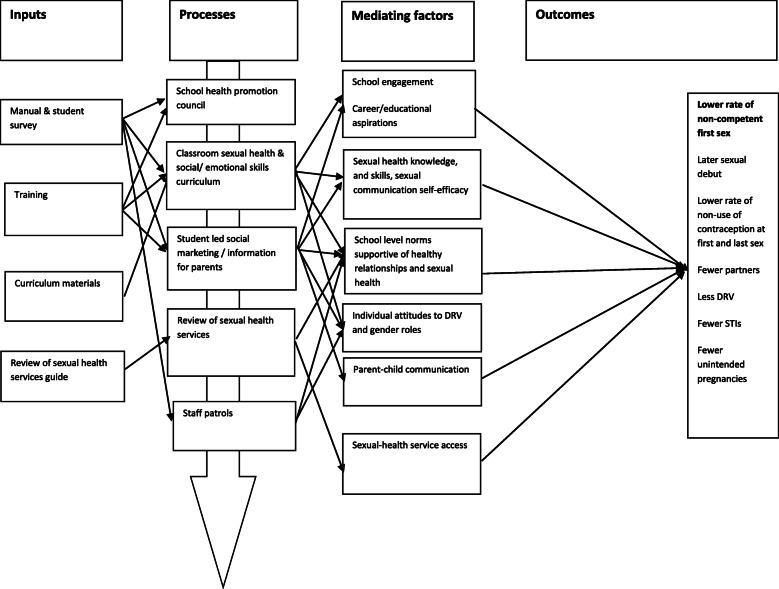
Logic model of Positive Choices

Positive Choice’s programme theory (Fig. 3) is informed by social marketing and has been developed with experts in this field, addressing the 4 ‘P’s [59, 60], ‘selling’ consumers a *P*roduct *they* want (holistic RSE based on expressed consumer needs) in an accessible *P*lace (school) at a low *P*rice (free to students), with *P*romotion by and to peers and to parents (campaigns, parent information), addressing competing influences from peers, media, etc. [61]. Positive Choices is also informed by social influence [62] and social cognitive theories [63] to address the following determinants of sexual and reproductive health including DRV: relationships/sexual health-related knowledge, skills and communication self-efficacy; attitudes about gender and DRV; social norms about healthy relationships (all via the curriculum and social marketing); and sexual health communication with parents (via parent information). It is also informed by the social development model [64], with student participation in school health promotion councils and social marketing campaigns theorised to increase school engagement, and positive career and educational aspirations [65], which are associated with better sexual health [66]. Review of school-based and local sexual and reproductive health services is theorised to improve access in line with NICE guidance [67].

Control schools will continue with existing RSE/sexual health provision, to be described via the process evaluation to maximise the external validity of the trial to assessing the intervention against normal provision in schools in England. Our pilot reported that schools varied in what was delivered but did not offer interventions components comparable to Positive Choices. Control schools will be informed by new statutory guidance on relationships, sex and health education. Compared to this statutory guidance, Positive Choices is more comprehensive in its coverage and much more focused on developing skills and self-efficacy, etc. and not merely knowledge. The new statutory guidance refers to whole-school approaches but does not suggest specific activities whereas Positive Choices includes school health promotion councils, needs surveys, student-run social marketing and review of sexual and reproductive health services. Hence, there will be a very clear distinction between intervention and comparator.

### Outcome measures

These will be assessed via self-reports at 33 months (age 15/16). All of our outcome measures have been piloted successfully in our pilot RCT and other studies (ordinal alphas for multi-item scales reported below) [42, 52].

Our primary outcome will be a binary measure of non-competent first sex. This measure has been chosen because it provides the best measure of overall sexual health in this age group regardless of gender or sexual orientation; is strongly associated with multiple adverse sexual health outcomes including STIs, non-volitional sex, unplanned pregnancy and sexual dysfunction [2]; was recommended through PPI with policy stakeholders and young people; is appropriate given the intervention now includes DRV prevention and is sufficiently prevalent among trial participants to provide statistical power through a pragmatic sample size [1, 2]. It has been recommended as an impact measure by the World Health Organization, is sensitive to intervention effects and is starting to be used in evaluations [68, 69]. Non-competent first sex will be assessed among trial participants having sex for the first time between baseline and follow-up, using the established Natsal self-report measure defined in terms of the absence at first sex of autonomy of decision; equal willingness of partners; its being the ‘right time’ and, for those reporting heterosexual intercourse, use of effective contraception [1, 2]. We will assess non-competent first sex for those reporting only same-sex intercourse via the other items but not assessing protection because this is primarily relevant for anal sex, which is extremely rare among this age group and according to our PPI is unacceptable to include in school surveys [70]. This primary outcome replaces pregnancy, which was the indicative primary outcome in the pilot RCT, because pregnancy was judged too narrow a measure of sexual health; pregnancy is relevant only for girls and heterosexual sex; pregnancy does not reflect the areas of greatest sexual health need in terms of current trends; and powering a trial on this outcome would require an unfeasibly large school sample of > 100 schools per arm [46]. Non-competence at first sex strongly predicts a range of subsequent adverse sexual health outcomes listed above, more so than is the case for other measures of early sexual risk behaviour [1, 2]. The pilot RCT indicated good performance of the Natsal competence measure in terms of completion (88%) and inter-item reliability (ordinal alpha = 0.74).

Secondary outcomes will examine other important potential benefits:

- Non-competent last sex [1, 2];
- Age at sexual debut, using an adapted version of the RIPPLE measure [71];
- Non-use of contraception at first and last sex among those reporting heterosexual intercourse, using an adapted version of the RIPPLE measure [71];
- Number of sexual partners, using an adapted RIPPLE measure [71];
- Dating and relationship violence (DRV) victimisation (including non-volitional sex) measured using an adapted version of the short Conflicts in Adolescent Dating Relationships Inventory (sCADRI) scale which is widely used and was piloted for reliability in our recent Project Respect study (ordinal alpha = 0.89) [42];
- Sexually transmitted infections (STIs), focused on self-reported diagnosis with common infections, using an adapted version of the RIPPLE measure [71];
- Pregnancy and unintended pregnancy for girls and initiation of pregnancy for boys using adapted versions of the RIPPLE measures [71].

#### Intermediate outcome measures

We will not undertake mediation analysis because this requires two waves of follow-up, which is not planned in the interests of minimising costs and maximising school retention. It is our experience across studies that multiple follow-ups reduce rather than maintain school engagement and hence response rates [51]. However, informed by our theory of change [63, 64], we will undertake exploratory analyses of intervention effects on the following ‘intermediate’ outcomes [72] using existing measures [36–39, 50, 73] tested for reliability in our pilot (ordinal alphas indicated below for multi-item scales): school-level social norms supportive of healthy relationships and sexual health (alpha = 0.77); individual-level attitudes towards DRV (alpha = 0.65) and gender roles (alpha = 0.68); individual-level school engagement (alpha = 0.74); career/educational aspirations; sexual health knowledge (alpha = 0.78); sexual health and contraceptive skills (alpha = 0.92); sexual communication self-efficacy (alpha = 0.93); and communication with parents; sexual and reproductive health services access (alpha = 0.83) [74].

#### Economic outcomes

Outcomes for the economic analysis will include the above primary and secondary outcomes. In addition, the Child Health Utility (CHU) 9D measure [75] will be used to assess student’s health-related quality of life. The CHU-9D is a validated age-appropriate measure that was explicitly developed using children’s input and has been suggested to be more appropriate and function better than other health utility measures for children and adolescents [76]. This measure performed well in the pilot. Student utility values will be collected at baseline and at follow-up surveys at 33 months using the CHU-9D questionnaire.

### Assessment and follow-up

Baseline surveys will be done before randomisation when students are in year 8 (age 12/13) in November 2021–March 2022 and will collect data on socio-demographic characteristics, sexual debut and other covariates, drawing on existing survey items [77]. Consent procedures are described under ethics below. Paper questionnaires will be completed confidentially in classrooms supervised by trained fieldworkers, with teachers remaining at the front of the class to maintain quiet and order, but unable to see student responses. Previous experience indicates that paper questionnaires are acceptable and logistically more straightforward than tablet surveys. We will survey absent students by leaving questionnaires and stamped addressed envelopes with schools, and liaising with schools to maximise returns. If schools do not allow face-to-face fieldwork visits, we will instead survey students using an online survey. This will involve students completing online surveys in classrooms supervised by teachers but with teachers remaining at the front of the class to maintain quiet and order, but unable to see student responses, and with fieldworkers available via telephone or internet technology to answer questions in real time. We will resurvey students at 33 months (September–December 2024) as students begin year 11 (age 15/16) and will collect self-report data on socio-demographic characteristics, intervention reach/acceptability and outcomes. Survey fieldworkers and analysts, but not students, will be blind to allocation. Based on past studies and our pilot [50, 71, 78], we expect at least 80% survey response rates at baseline and follow-up.

### Process evaluation data collection

Integral process evaluation informed by existing frameworks [79–82] will examine: intervention fidelity, reach and acceptability and how this varies by school and student; usual treatment in control schools and potential contamination; and implementation processes/intervention mechanisms and how these vary between schools and students.

#### Intervention fidelity, reach, acceptability and context

Fidelity of implementation of all intervention components by schools and preparatory activities (start-up meetings; training) by SEF will be assessed quantitatively using bespoke measures developed in the pilot RCT. Data will be collected via audio-recording of SEF training for school staff; surveys of school staff trained by SEF; logbooks of school staff implementing internal training, school health promotion councils, curriculum and social marketing meetings; and structured researcher observations of one randomly selected session per school of internal training, school health promotion councils, curriculum lessons and social marketing meetings. Across all schools, the RSE coordinator will complete one logbook summarising delivery across the school and teachers will complete one logbook summarising whether the key points of each lesson were taught. In the four case study schools (see below), logbooks will examine delivery of the contents of each lesson in more detail. This approach is informed by the finding from the pilot RCT that collecting detailed information on delivery of every lesson from every teacher is not feasible or acceptable to teachers. Observations will act as a check on the reliability of data from logbooks. We will primarily assess fidelity of form (i.e. of activities) but where local adaptations are made we will assess whether these are consistent or not with intervention theory of change in order to provide a qualitative assessment of fidelity of function (i.e. to theory of change) [83]. We will examine reach and acceptability to students (overall and by student gender, sexual orientation, ethnicity and SES, and by school-level GCSE attainment and local deprivation) quantitatively via questionnaire survey items at follow-up.

#### Comparator and potential contamination

We will examine RSE and sexual and reproductive health service provision in and around control schools in order to describe usual treatment. We will examine the potential for contamination across arms to assess whether this is a threat to internal validity. Data will be collected via student surveys and structured phone interview with 1 staff member (using the School Health Research Network school health questionnaire [84]) per control school per year.

#### Implementation processes/intervention mechanisms and context

Informed by May’s implementation theory and realist evaluation [80, 82], we will collect qualitative data and analyse these in order to explore implementation processes and intervention mechanisms (beneficial or harmful) and how these vary between schools and students. Data will be collected from all intervention schools via phone interview with one staff member and face-to-face focus group with 4–8 students per school (purposive by involvement and gender) per year of delivery. More in-depth data will be collected from 4 case study schools purposively sampled by GCSE attainment and local deprivation via phone interview with 4 staff members and 2 face-to-face focus groups with 4–8 students (purposive by involvement and gender and ethnicity) per year of delivery. It will not be feasible in schools to purposively sample students by sexual orientation or socioeconomic status but we will strive to be inclusive of a diversity of students.

### Economic evaluation data collection

Our economic measure of child health-related quality of life (CHU-9D) is described above [75]. We will undertake a detailed micro-costing of the the intervention, which was found to be feasible in the pilot RCT, including the costs of all components of the intervention described above. Resources to be measured will include resources used by SEF and schools in terms of staff time, training events/meetings and consumables. Measures will include standardised sessional checklists to monitor and document attendance, preparation and delivery time for key training events, school health promotion councils, student-run social marketing meetings and the review of school sexual and reproductive health services; the completion of surveys and diaries by school staff charged with training, curriculum and other intervention delivery; assessing time spent on tasks relating to intervention; and staff travel and other expenses relating to the intervention charged to a specific SEF budget code. Resource use will be valued using market prices. We will also include the costs within the trial period arising from primary and secondary outcomes (valued from published sources, obtained from systematic literature searches), in particular pregnancy, STIs and DRV-related costs, where these are shown to be different between the intervention and control groups. Costs will be presented for each of the perspectives described above.

### Data management and analysis

Anonymised survey data will be managed by LSHTM’s accredited CTU with linkage to unique identifier codes (not names) in password-protected files on drives accessible only by named CTU staff. The fieldwork team will manage a separate data-file linking names to unique identifiers, in similarly protected files and drives, and will not have access to self-report survey data. This will maintain separation of identifiers and self-report data. Audio-recordings will use secure password-protected recorders. These will be transcribed in full by LSHTM-approved contractors with secure data transfer and management processes. Transcripts will be anonymised and stored in secure files and drives by the fieldwork team. All reporting will be fully anonymised to prevent explicit or implied identification. In line with MRC guidance on personal information in medical research, we will retain all anonymised research data for 20 years after the end of the study. This is to allow secondary analyses and further research to take place, and to allow any queries or concerns about the conduct of the study to be addressed.

A statistical analysis plan will be drafted by the CTU and a data dictionary by the fieldwork team. Statistical analysis of survey data will be led by the CTU, with analysis of fidelity data by the fieldwork team and economic data by Professor Morris. School randomisation and retention, and student response rates will be described using a CONSORT diagram [85]. Baseline and follow-up data will be tabulated by arm. Continuous outcomes will be summarised for each trial arm using means and standard deviations and binary outcomes will be summarised for each trial arm using numbers and percentages. We will conduct intention-to-treat analyses of primary and secondary outcomes [85] as well as exploratory analyses of our intermediate outcomes (RQ1) to estimate intervention effects and 95% confidence intervals. The analytic sample will be students providing follow-up data at 33 months. Analyses will account for school clustering. For binary outcomes, we will use mixed-effect logistic regression with a random effect for school, reporting odds ratios (OR). For continuous outcomes, we will use multi-level linear mixed models (with a random effect for school), reporting mean differences. We will present unadjusted analyses plus analyses adjusting for school-level stratifying variables, and baseline student age, gender, ethnicity and SES (as measured by the family affluence scale) and age of sexual debut, these being imputed from follow-up data when missing at baseline. Interim analyses will not be conducted and will not inform intervention modification or discontinuation because this is a cluster RCT with one follow-up wave.

We will undertake exploratory tests of interaction to assess how effects are moderated, including by but not exclusive to student gender, sexual orientation, ethnicity and SES as well as by school GCSE attainment and local deprivation (RQ2). These will be exploratory, with limited statistical power. We will also undertake on-treatment analyses to examine how effects are affected by fidelity of implementation (RQ3). Economic analyses will calculate intervention costs and cost-effectiveness (RQ4) (see below). Descriptive statistics will describe fidelity, reach and acceptability using chi-square tests to examine differences between schools. These will examine whether fidelity differs by school-level GCSE attainment and local deprivation, and whether student-level reach and acceptability differ by student-level gender, sexual orientation, ethnicity and SES (RQ5). Quantitative and qualitative data from control schools will be analysed to describe usual treatment in control schools (RQ6). Qualitative data will also be used to develop hypotheses about implementation processes/intervention mechanisms and how these might vary between schools or students (RQ7). We will draw on all data to refine the intervention theory of change and draw conclusions about the potential for the intervention to be delivered and be effective elsewhere (RQ8).

#### Economic analyses

The aim is to evaluate the cost-effectiveness of Positive Choices versus the comparator from a health and social care perspective as preferred by NICE, additionally considering education and voluntary-sector perspectives [86]. An economic analysis plan will be drafted before receipt of the final data. For our primary cost-utility analysis, costs and CHU-9D data will be combined to present mean cost and quality-adjusted life-years and incremental cost-effectiveness ratios. A secondary cost-consequences analysis will additionally be undertaken for the other trial outcomes as recommended by NICE public health methods guidance [87] because there may not be a strong correlation between CHU-9D and these other outcomes. We will use a multi-level modelling approach with random intercepts to estimate the mean and standard errors for both cost and effects along with the covariance matrix [88]. From these, mean incremental net benefit and confidence intervals will then be estimated. Missing data will be handled using multiple imputation [89]. The time horizon will capture costs and outcomes within the trial. The pilot RCT indicated these analyses as feasible.

*Qualitative data* will be subject to thematic content analysis (in vivo/axial codes; constant comparison [90]) informed by realist approaches to evaluation [82] and May’s implementation theory [80] to examine implementation processes, potential intervention mechanisms and how these vary between schools and students. Realist analyses of qualitative data will inform refinements to our theory of change and, where possible, inform additional hypotheses about how context interacts with intervention mechanisms to generate outcomes. These analyses and hypotheses will be posted online prior to assessing them via post hoc exploratory mediation and moderation analyses of quantitative data. In the light of these analyses, we will refine our intervention theory of change, defining what contextual factors promote or impede implementation and mechanisms. This refined theory of change will be the means by which we will make evidence-informed suggestions about the potential for the intervention to be delivered effectively elsewhere.

### Project oversight

The study SSC and DMEC will be independent from the research team and sponsor, appointed by the funder with respectively oversight and audit of trial design and conduct, and of data integrity, ethics and participant safety. Composition is available on request.

### Public and policy involvement

The phase III study will continue to collaborate with the ALPHA and policy-practice stakeholder groups. These are discussed above, integrated into our description of methods. Meetings with each of these groups near the start of the study will critically appraise drafts of the new intervention materials prior to their finalisation. A meeting with each of these groups near the end will focus on interpretation and dissemination of our results and the potential for scale-up of the intervention. We include letters of support from organisations indicating that they are already willing to participate in this group. We will also invite a teacher and two students from schools in the pilot RCT to sit on the SSC so that this takes on a broader perspective.

### Ethical issues, safeguarding and SAEs

All participants will be informed in consent materials that the information they provide will be treated with anonymity and confidentiality, as well as the circumstances in which we would need to breach confidentiality. We will maintain standard operating procedures for dealing with safeguarding concerns. In the pilot, we worked with a child protection social worker to develop a priori categories of abuse reported through the research that would necessitate our breaching confidentiality to ensure individuals are offered care and protection. These are informed by existing clinical guidelines and for example include sex before age 13 as well as forced sex or other serious abuse. These categories balance our ethical duty of promoting participant autonomy and wellbeing. Where defined categories of abuse are indicated in questionnaires, we will contact the safeguarding lead in the school. Where these are reported directly to research staff during data collection, we will first discuss the need for a response with the individual prior to contacting the school safeguarding lead. In each school and within NCB, a senior member of staff will be identified who is not directly involved with the intervention and to whom staff or students may go if they have complaints about any elements of the intervention or research.

Interviews, focus groups and observations will not aim to explore experiences of sex or abuse. In the case of focus groups, our researchers will be trained to ensure that discussions do not move in the direction of personal disclosures since this is not the purpose of the groups and it would be very difficult to ensure that other participants do not communicate such disclosures outside the group. However, if participants in interviews or focus groups describe abuse or become upset in any way, our researchers will be trained in how to respond. In interviews, researchers will stop the interview and determine need for a referral to support within the school. In focus groups, researchers will aim to stop sensitive discussions, and assess need for individual support at the end or stop the focus group if the assessment is that immediate support is needed.

We will monitor safeguarding concerns and standard categories of SAEs via regular consultation with schools. The SSC, data monitoring and ethics committee and LSHTM Ethics Committee will be provided with anonymised reports of safeguarding concerns and SAEs, categorised by type, circumstances and the plausibility that these are related to intervention or research activities. Because all follow-ups occur at 33 months, there will be no interim analyses. The DMEC will consider stopping if there is any suggestion of an association between the number of safeguarding concerns and SAEs plausibly associated with the intervention or trial and the arm of the trial.

## Discussion

Substantial population-level sexual health impacts, for example on teenage pregnancy and DRV, cannot be achieved via targeted interventions [19, 48]; therefore, Positive Choices is a universal intervention. Nonetheless, the intervention aims to maximally benefit disadvantaged and minority students by addressing the more upstream determinants of sexual health, such as engagement with school and gender and other social norms, and by ensuring that disadvantaged and minority students are involved in and reached by intervention activities such as school health promotion councils and social marketing campaigns. Research methods will also be inclusive for disadvantaged and minority students, for example using fieldworkers from diverse backgrounds, using plain written English materials and supporting all students who need help in surveys and other data collection. Our primary outcome and most of our secondary outcomes are inclusive with regard to gender and sexual orientation. We will assess how intervention reach, acceptability and effects vary by student gender, sexual orientation, ethnicity and SES, and by school-level measures of GCSE attainment and local deprivation. Student recruitment to qualitative research will be purposive by gender and ethnicity. It will not be feasible in schools to purposively sample students by sexual orientation or socioeconomic status but we will strive to be inclusive of a diversity of students.

To maximise external validity, the study will aim to recruit a broadly representative sample of secondary schools in central and southern England. To maintain the integrity of the intervention and analyses, the fieldwork, intervention and CTU teams will be separately managed. To minimise confounding and bias, the evaluation design will be experimental with random allocation of schools not individual students (to minimise contamination and preserve the school-level theory of change) stratified by school-level GCSE attainment and local index of deprivation by the CTU after baseline surveys. To minimise retention bias, we will maximise school retention using the methods described above, including school payments. To minimise analytic bias, we will publish a study protocol including analytic plans which clearly distinguish between primary, secondary and exploratory analyses. Furthermore, quantitative data will be collected and analysed blind to allocation (blinding of participants is not possible) with primary analyses being intention-to-treat focused on valid and reliable outcome measures adjusting for potential baseline imbalances. Our analytical sample will be those students providing data at follow-up with imputation of missing baseline data on socio-demographic factors and age of sexual debut based on responses at follow-up. Quantitative analyses will be undertaken by statisticians from LSHTM’s accredited CTU staff, with oversight by the DMEC. To minimally bias thematic analysis, qualitative analyses will be undertaken prior to quantitative analyses. To minimise ‘data dredging’ within exploratory analyses, we will ensure these focus on a small number of post hoc hypotheses that have been developed informed by our qualitative analyses and posted online prior to quantitative assessment.

Our knowledge exchange will be informed by consultation at the end of the study with the ALPHA young researchers’ group as well as with our group of policy/practitioner stakeholders. As well as reporting in the NIHR Public Health Research journal, we will submit two open-access papers to top journals. Authorship will follow International Committee of Medical Journal Editors guidance. We will present our findings at two international conferences (Society of Prevention Research; International Association for Adolescent Health), plus national academic and policy conferences. We will disseminate the results to participating schools, the ALPHA group and the policy/practice stakeholder group. We will draft an article for the Times Education Supplement about the research. The research team will also use blog-posts and Twitter to increase public awareness of the study. A policy and practice dissemination event will be held at SEF targeting the central and local government, education, public health and voluntary sectors. The most important scientific outputs generated by this project will be evidence about the effectiveness, costs and potential scalability and transferability of a whole-school social marketing intervention to promote sexual health. If this trial finds that Positive Choices is effective in reducing non-competent first sex, this would be scaled up by SEF working collaboratively with the investigators, marketing the intervention to secondary schools, local authorities, school networks and academy chains who would be charged by SEF to be trained to deliver the intervention. Background intellectual property for the intervention lies with LSHTM. Foreground intellectual property for additional intervention materials and the outputs of the research will lie with LSHTM, which will grant the collaborating institutions licence to use these materials in teaching and training activities. Licence to use the intervention materials in scaled up delivery will be contingent on the evaluated effectiveness of the intervention.

### Trial status

Schools will be recruited September–December 2021 and students will be recruited and surveyed November 2021–March 2022.

## Data Availability

Data will be made available after the main trial analyses have been completed on reasonable request from researchers with ethics approval and a clear protocol.

## Abbreviations

ALPHA: Advice Leading to Public Health Action
CHU9D: Child Health Utility 9D measure
CONSORT: Consolidated standards of reporting trials
CTU: Clinical trials unit
DECIPHer: Centre for Development, Evaluation, Complexity and Implementation in Public Health Improvement
DMEC: Dating monitoring committee
DRV: Dating and relationship violence
GCSE: General Certificate of Secondary Education
ICC: Intra-cluster correlation coefficient
IDACI: Income Deprivation Affecting Children Index
LSHTM: London School of Hygiene and Tropical Medicine
Natsal: National Survey of Sexual Attitudes and Lifestyles PPI Patient and policy involvement
NHS: National Health Services
NICE: National Institute for Health and Clinical Excellence
Ofsted: Office for Standards in Education, Children’s Services and Skills
OR: Odds ratio
PSHE: Personal, Social, Health and Economic Education
RCT: Randomised controlled trial
RIPPLE: Randomised intervention for pupil-led sex education in England
RQ: Research question
RSE: Relationships and sex education
SAE: Serious adverse event
sCADRI: Short Conflicts in Adolescent Dating Relationships Inventory
SEF: Sex Education Forum
SES: Socioeconomic status
SSC: Study steering committee
STI: Sexually transmitted infections
UK: United Kingdom

## References

[1] Palmer MJ, Clarke L, Ploubidis GB, Wellings K (2019). Prevalence and correlates of 'sexual competence' at first heterosexual intercourse among young people in Britain. BMJ Sexual and Reproductive Health..

[2] Palmer MJ, Clarke L, Ploubidis GB, Mercer H, Gibson LJ, Johnson AM (2017). Is “sexual competence” at first heterosexual intercourse associated with subsequent sexual health status?. Journal of Sex Research..

[3] Lara LA, Abdo CHN (2016). Age at time of initial sexual intercourse and health of adolescent girls. Journal of Pediatric and Adolescent Gynecology.

[4] Lewis R, Tanton C, Mercer CH, Mitchell KR, Palmer M, Macdowall W, Wellings K (2017). Heterosexual practices among young people in Britain: evidence from three national surveys of sexual attitudes and lifestyles. Journal of Adolescent Health..

[5] England PH (2019). Sexually transmitted infections and screening for chlamydia in England. Health Protection Report.

[6] Department of Health and Social Care (2019). Abortion statistics for England and Wales: 2018.

[7] Macdowall W, Gibson LJ, Tanton C, Mercer CH, Lewis R, Clifton S, Field N, Datta J, Mitchell KR, Sonnenberg P, Erens B, Copas AJ, Phelps A, Prah P, Johnson AM, Wellings K (2013). Lifetime prevalence, associated factors, and circumstances of non-volitional sex in women and men in Britain: findings from the third National Survey of Sexual Attitudes and Lifestyles (Natsal-3). Lancet..

[8] Sonnenberg P, Clifton S, Beddows S, Field N, Soldan K, Tanton C, Mercer CH, da Silva FC, Alexander S, Copas AJ, Phelps A, Erens B, Prah P, Macdowall W, Wellings K, Ison CA, Johnson AM (2013). Prevalence, risk factors, and uptake of interventions for sexually transmitted infections in Britain: fi ndings from the National Surveys of Sexual Attitudes and Lifestyles (Natsal). Lancet..

[9] Wellings K, Jones KG, Mercer CH, Tanton C, Clifton S, Datta J, Copas AJ, Erens B, Gibson LJ, Macdowall W, Sonnenberg P, Phelps A, Johnson AM (2013). The prevalence of unplanned pregnancy and associated factors in Britain: findings from the third National Survey of Sexual Attitudes and Lifestyles (Natsal-3). Lancet..

[10] Barter C, Aghtaie N, Larkins C (2014). Safeguarding Teenage Intimate Relationships (STIR). Connecting online and offline contexts and risks. Briefing Paper 2: Incidence Rates and Impact of Experiencing Interpersonal Violence and Abuse in Young People’s Relationships.

[11] Decker MR, Silverman JG, Raj J (2005). Dating violence and sexually transmitted disease/HIV testing and diagnosis among adolescent females. Pediatrics..

[12] Exner-Cortens D, Eckenrode J, Rothman E. Longitudinal associations between teen dating violence victimization and adverse health outcomes. Pediatrics. 2013;131(71):e8, 71, 78, DOI: 10.1542/peds.2012-1029. 10.1542/peds.2012-1029 PMC3529947 23230075

[13] Office for National Statistics (2019). Conceptions in England and Wales: 2017.

[14] Ashcraft A, Fernández-Val I, Lang K (2013). The consequences of teenage childbearing: consistent estimates when abortion makes miscarriage non-random. The Economic Journal..

[15] Fletcher J, Wolfe B (2009). Education and labor market consequences of teenage childbearing: evidence using the timing of pregnancy outcomes and community fixed effects. Journal of Human Resources..

[16] Ermisch J (2003). Does a ‘teen-birth’ have longer-term impacts on the mother? Suggestive Evidence from the British Household Panel Survey.

[17] Berthoud R, Ermisch J, Francesconi M, Liao T, Pevalin D, Robson K (2004). Long-Term Consequences of teenage births or parents and their children. Teenage Pregnancy Research Programme Research Briefing, No. 1.

[18] Francesconi M. Adult outcomes for children of teenage mothers. Scand J Econ. 2008;110(93):e117, 93, 117, DOI: 10.1111/j.1467-9442.2008.00526.x.

[19] Kneale D, Fletcher A, Wiggins R, Bonell C (2013). Distribution and determinants of risk of teenage-motherhood in three British longitudinal studies: implications for targeted prevention interventions. Journal of Epidemiology and Community Health..

[20] Family Planning Association and Brook (2013). Unprotected nation: the finanacial and economic impacts of restricted contraceptive and sexual health services.

[21] Public Health England. Contraception: economic analysis estimation of the return on investment (ROI) for publicly funded contraception in England London: PHE; 2018.

[22] DiCenso A, Guyatt G, Willan A, Griffith L (2002). Interventions to reduce unintended pregnancies among adolescents: systematic review of randomised controlled trials. British Medical Journal..

[23] Kirby D (2007). Emerging Answers 2007: research findings on programs to reduce teen pregnancy and sexually transmitted diseases.

[24] Mason-Jones AJ, Sinclair D, Mathews C, Kagee A, Hillman A, Lombard C. School-based interventions for preventing HIV, sexually transmitted infections, and pregnancy in adolescents. Cochrane Database of Systematic Reviews 2016(11):Art. No.: CD006417. DOI: 10.1002/14651858.CD006417.pub3. 10.1002/14651858.CD006417.pub3 PMC5461872 27824221

[25] Downing J, Jones L, Cook P, Bellis M (2006). Prevention of sexually transmitted infections (STIs): a review of reviews into the effectiveness of non-clinical interventions: Evidence Briefing Update.

[26] Oringanje C, Meremikwu MM, Eko H (2009). Interventions for preventing unintended pregnancies among adolescents. Cochrane Database of Systematic Reviews..

[27] Shepherd J, Kavanagh J, Picot J, Cooper K, Harden A, Barnett-Page E, Jones J, Clegg A, Hartwell D, Frampton GK, Price A (2010). The effectiveness and cost-effectiveness of behavioural interventions for the prevention of sexually transmitted infections in young people aged 13 to 19: a systematic review and economic evaluation. Health Technol Assess Monographs.

[28] Shackleton N, Jamal F, Viner RM, Dickson K, Patton G, Bonell C (2016). School-level interventions going beyond health education to promote adolescent health: systematic review of reviews. Journal of Adolescent Health..

[29] Haberland NA (2015). The case for addressing gender and power in sexuality and HIV education: a comprehensive review of evaluation studies. International Perspectives on Sexual and Reproductive Health..

[30] Blank L, Baxter SK, Payne N, Guillaume LR, Pilgrim H (2010). Systematic review and narrative synthesis of the effectiveness of contraceptive service interventions for young people, delivered in educational settings. Journal of Pediatric and Adolescent Gynecology..

[31] Harden A, Brunton G, Fletcher A, Oakley A (2009). Teenage pregnancy and social disadvantage: a systematic review integrating trials and qualitative studies. British Medical Journal..

[32] Peterson AJ, Donze M, Allen E, Bonell C (2018). Effects of interventions addressing school environments or educational assets on adolescent sexual health: systematic review and meta-analysis. International Perspectives on Sexual and Reproductive Health..

[33] Salam RA, Faqqah A, Sajjad N, Lassi ZS, Das JK, Kaufman M, et al. Improving adolescent sexual and reproductive health: a systematic review of potential interventions. Journal of Adolescent Health. 2016;59(4 (Suppl)):S11-S28. 10.1016/j.jadohealth.2016.05.022 PMC5026684 27664592

[34] Andreasen AR (2002). Marketing social marketing in the social change marketplace. Journal of Public Policy and Marketing..

[35] Simiyu Wakhisi A, Allotey P, Dhillon N, Reidpath DD (2011). The effectiveness of social marketing in reduction of teenage pregnancies: a review of studies in developed countries. Social Marketing Quarterly..

[36] Basen-Engquist K, Coyle K, Parcel GS, Kirby D, Banspach SW, Carvajal SC (2009). School wide effects of a multicomponent HIV, STD and pregnancy prevention program for high school students. Health Education and Behavior..

[37] Coyle K, Basen-Engquist K, Kirby D, Parcel G, Banspach S, Collins J, Baumler E, Carvajal S, Harrist R (2001). Safer choices: reducing teen pregnancy, HIV, and STDs. Public Health Reports..

[38] Coyle K, Basen-Engquist K, Kirby D, Parcel G, Banspach S, Harrist R, Baumler E, Weil M (1999). Short-term impact of safer choices: a multicomponent, school-based HIV, other STD, and pregnancy prevention program. J Sch Health..

[39] Philliber S, Kaye JW, Herrling S, West E (2002). Preventing pregnancy and improving health care access among teenagers: an evaluation of the Children’s Aid Society-Carrera Program. Perspectives on Sexual and Reproductive Health..

[40] Patton G, Bond L, Carlin JB, Thomas L, Butler H, Glover S (2006). Promoting social inclusion in schools: group-randomized trial of effects on student health risk behaviour and well-being. Am J Public Health..

[41] Wiggins M, Bonell C, Sawtell M, Austerberry H, Burchett H, Allen E, Strange V (2009). Health outcomes of youth development programme in England: prospective matched comparison study. British Medical Journal..

[42] Meiksin R, Crichton J, Dodd J, Morgan GS, Williams P, Willmott M (2020). Project Respect: pilot cluster RCT of a school-based intervention to prevent dating and relationship violence among young people. Public Health Research.

[43] Wolfe DA, Crooks C, Jaffe P, Chiodo D, Hughes R, Ellis W, Stitt L, Donner A (2009). A school-based program to prevent adolescent dating violence: a cluster randomized trial. Archives of Pediatric and Adolescent Medicine..

[44] Foshee VA, Bauman KE, Ennett ST, Linder GF, Benefield T, Suchindran C (2004). Assessing the long-term effects of the Safe Dates program and a booster in preventing and reducing adolescent dating violence victimization and perpetration. American Journal of Public Health..

[45] Taylor BG, Stein ND, Mumford E, Woods D (2013). Shifting Boundaries: An experimental evaluation of a dating violence prevention program in middle schools. Prevention Science.

[46] Office for National Statistics (2019). Statistical bulletin: conceptions in England and Wales: 2016 Annual statistics on conceptions to residents of England and Wales; numbers and rates, by age group including women aged under 18 years.

[47] Philiber S, Kaye JW, Herrling S (2001). The National Evaluation of the Children’s Aid Society Carrera Model Program to Prevent Teen Pregnancy.

[48] Crawford C, Cribb J, Kelly E (2013). Teenage Pregnancy in England CAYT Impact Study: Report No. 6.

[49] Henderson M, Wight D, Raab G, Abraham C, Parkes A, Scott S (2007). ‘Impact of a theoretically based sex education programme (SHARE) delivered by teachers on NHS registered conceptions and terminations: final results of a cluster randomised trial. British Medical Journal..

[50] Stephenson JM, Strange V, Forrest S, Oakley A, Copas A, Allen E, Babiker A, Black S, Ali M, Monteiro H, Johnson AM (2004). Pupil-led sex education in England (RIPPLE study): cluster-randomised intervention trial. Lancet..

[51] Bonell C, Allen E, Warren E, McGowan J, Bevilacqua L, Jamal F, Legood R, Wiggins M, Opondo C, Mathiot A, Sturgess J, Fletcher A, Sadique Z, Elbourne D, Christie D, Bond L, Scott S, Viner RM (2018). Initiating change in the school environment to reduce bullying and aggression: a cluster randomised controlled trial of the Learning Together (LT) intervention in English secondary schools. The Lancet..

[52] Ponsford R, Bragg S, Allen E, Tilouche N, Meiksin R, Emmerson L (2021). A school-based social-marketing intervention to promote sexual health in English secondary schools. the Positive Choices pilot cluster RCT Public Health Research.

[53] Department for Education (2019). Schools, Pupils and their Characteristics: January 2019.

[54] Inchley J, Currie D, Young T, Samdal O, Torsheim T, Augustson L (2016). Growing up unequal: gender and socioeconomic differences in young people’s health and well-being. Health Behaviour in School-Aged Children (HBSC) Study: International Report From the 2013/1014 Survey.

[55] Parkes A, Wight D, Henderson M, Stephenson J, Strange V (2009). Contraceptive method at first sexual intercourse and subsequent pregnancy risk: findings from a secondary analysis of 16-year-old girls from the RIPPLE and SHARE studies. Journal of Adolescent Health..

[56] Brooks F, Magnusson J, Klemera E, Chester K, Spencer N, Smeeton N (2015). HBSC England National Report 2014.

[57] Lohan M, Aventin Á, Maguire L, Curran R, McDowell C, A. A, et al. Increasing boys' and girls' intention to avoid teenage pregnancy: a cluster randomised control feasibility trial of an interactive video drama based intervention in post-primary schools in Northern Ireland. Public Health Research. 2017;5(1). 28358459

[58] Fletcher A, Fitzgerald-Yau N, Wiggins M, Viner R, Bonell C. Involving young people in changing their school environment to make it safer: findings from a process evaluation in English secondary schools. Health Education (Special Issue on Health Promoting Schools). 2015;(in press).

[59] Hastings G, McDermott L (2006). Putting social marketing into practice. British Medical Journal..

[60] Hastings G, Stead M, Macdowall M, Bonell C, Davies M (2006). Social Marketing. Health Promotion Practice.

[61] Fletcher A, Harden A, Brunton G, Oakley A, Bonell C (2008). Interventions addressing the social determinants of teenage pregnancy. Health Education..

[62] Fisher JD (1988). Possible effects of reference group-based social influence on AIDS-risk behaviors and AIDS. Am Psychol.

[63] Bandura A (1986). Social foundations of thought and action: a social cognitive theory.

[64] Hawkins JD, Weiss JG (1985). The social development model: an integrated approach to delinquency prevention. Journal of Primary Prevention..

[65] Gavin LE, Catalano RF, David-Ferdon C, Gloppen KM, Markham CM (2010). A review of positive youth development programs that promote adolescent sexual and reproductive health. Journal of Adolescent Health..

[66] Bonell C, Allen E, Strange V, Copas A, Oakley A, Stephenson J, Johnson A (2005). The effect of dislike of school on risk of teenage pregnancy: testing of hypotheses using longitudinal data from a randomised trial of sex education. J Epidemiol Community Health..

[67] National Institute for Health and Clinical Excellence (2014). Contraceptive services with a focus on young people aged up to 25 overview.

[68] Forsyth R, Purcell C, Barry S, Simpson S, Hunter R, McDaid L, Elliot L, Bailey J, Wetherall K, McCann M, Broccatelli C, Moore L, Mitchell K (2018). Peer-led intervention to prevent and reduce STI transmission and improve sexual health in secondary schools (STASH): protocol for a feasibility study. Pilot and Feasibility Studies.

[69] World Health Organization (2010). Measuring sexual health: conceptual and practical considerations and related indicators.

[70] Weatherburn P, Hickson S, Reid DS, Schink SB, Marcus U, Schmidt AJ (2019). European Men-Who-Have-Sex-With-Men Internet Survey (EMIS-2017): Design and Methods. EMIS-2017 – The European Men-Who-Have-Sex-With-Men Internet Survey: key findings from 50 countries.

[71] Stephenson J, Strange V, Allen E, Copas A, Johnson A, Bonell C, Babiker A, Oakley A, the RIPPLE Study Team The long-term effects of a peer-led sex education programme (RIPPLE): a cluster randomised trial in schools in England. PLoS Med. 2008;5(11):e224, e224; discussion e224, DOI: 10.1371/journal.pmed.0050224. 10.1371/journal.pmed.0050224 PMC2586352 19067478

[72] Baron RM, Kenny DA (1986). The moderator-mediator variable distinction in social psychological research: conceptual, strategic and statistical considerations. Journal of Personality and Social Psychology..

[73] Wellings K, Nanchahal K, Macdowall W, McManus S, Erens B, Mercer CH, Johnson AM, Copas AJ, Korovessis C, Fenton KA, Field J (2001). Sexual behaviour in Britain: early heterosexual experience. Lancet..

[74] AAUW (2001). Educational Foundation. Hostile hallways: bullying, teasing, and sexual harassment in school.

[75] Ware. J Jr. K, M. and Keller, SD. . A 12-item short-form health survey: construction of scales and preliminary tests of reliability and validity. Med Care. 1996;34(3):220-233, DOI: 10.1097/00005650-199603000-00003. 10.1097/00005650-199603000-00003 8628042

[76] Canaway AG, Frew EJ (2013). Measuring preference-based quality of life in children aged 6-7 years: a comparison of the performance of the CHU-9D and EQ-5D-Y--the WAVES pilot study. Quality of Life Research..

[77] Brooks F, Magnusson J, Klemera E, Spencer N, Morgan A. HBSC England National Report. Findings from the 2010 HBSC study for England. Hatfield: University of Hertfordshire; 2011.

[78] Bonell CP, Fletcher A, Fitzgerald-Yau N, Hale D, Allen E, Elbourne D, Jones R, Bond L, Wiggins M, Miners A, Legood R, Scott S, Christie D, Viner R (2015). Initiating change locally in bullying and aggression through the school environment (INCLUSIVE): pilot randomised controlled trial. Health Technology Assessment..

[79] Linnan L, Steckler A (2002). Process Evaluation for Public Health Interventions and Research.

[80] May C (2013). Towards a general theory of implementation. Implementation Science..

[81] Moore G, Audrey S, Barker M, Bond L, Bonell C, Hardeman W (2014). Process evaluation of complex interventions: UK Medical Research Council (MRC) guidance.

[82] Pawson R, Tilley N (1997). Realistic Evaluation.

[83] Hawe P, Shiell A, Riley T (2004). Complex interventions: how "out of control" can a randomised controlled trial be?. British Med Journal..

[84] Murphy S, Littlecott H, Hewitt G, MacDonald S, Roberts J, Bishop J, et al. A transdisciplinary complex adaptive systems (T-CAS) approach to developing a national school-based culture of prevention for health improvement: the School Health Research Network (SHRN) in Wales. Prevention Science doi: 101007/s11121-018-0969-3 [Epub ahead of print]. 2018. 10.1007/s11121-018-0969-3 PMC7762741 30536190

[85] Campbell MK, Piaggio G, Elbourne DR, Altman DG, for the CONSORT Group (2012). Consort 2010 statement: extension to cluster randomised trials. British Medical Journal.

[86] National Institute for Health and Care Excellence (2013). Guide to the Methods of Technology Appraisal.

[87] Nationale Institute for Health and Care Excellence (2012). Methods for the Development of NICE Public Health Guidance.

[88] Gomes M, Ng E, Grieve R, Nixon R, Carpenter JR, Thompson SG (2011). Developing appropriate methods for cost-effectiveness analysis of cluster randomized trials. Medical Decision Making..

[89] Gomes M, Diaz-Ordaz K, Grieve R, Kenward MG (2013). Multiple imputation methods for handling missing data in cost-effectiveness analyses that use data from hierarchical studies: an application to cluster randomized trials. Medical Decision Making..

[90] Green J, Thorogood N (2004). Qualitative Methods for Health Research.

